# Effectiveness of 2 Just-in-Time Adaptive Interventions for Reducing Stress and Stabilizing Cardiac Autonomic Function: Microrandomized Trials

**DOI:** 10.2196/69582

**Published:** 2025-08-07

**Authors:** Andreas Richard Schwerdtfeger, Josef Martin Tatschl, Christian Rominger

**Affiliations:** 1 Department of Psychology University of Graz Graz Austria

**Keywords:** cardiac vagal regulation, just-in-time adaptive intervention, JITAI, microrandomized trial, resonance breathing, mindful breathing

## Abstract

**Background:**

Heart rate variability (HRV) indicates brain-body interaction and has been associated with a variety of mental and physical health indicators. Transient reductions in HRV, independent of bodily movement (so-called additional HRV reduction [AddHRVr]), may reflect moments of psychophysiological vulnerability. This metric is quantified by regressing bodily movement on the root mean square of successive differences and identifying reductions <0.5 SD of the predicted value in real time in everyday life.

**Objective:**

We aimed to apply this measure using wearables in everyday life to trigger low-threshold, 1-minute just-in-time adaptive interventions (JITAIs) to stabilize autonomic function and relieve perceived stress and ruminative thoughts. Thus, we compared moments of AddHRVr with random points in time with respect to the effects of brief JITAIs.

**Methods:**

In 2 preregistered microrandomized trials, participants underwent a 1-day calibration period to derive individualized trigger settings and then received AddHRVr-triggered and random prompts throughout the following 3 days, asking for perceived stress and rumination. In study 1, participants (N=60) underwent a slow breathing intervention (0.1 Hz slow-paced breathing) following each prompt, and in study 2, participants (N=50) were microrandomized to both an external attention (ie, focusing attention on a nonliving object) and a mindful breathing intervention. HRV was assessed before, during, and following each intervention by means of the root mean squares of successive differences, SD of normal-to-normal beats, and high- and low-frequency HRV. Participants also reported on perceived stress and ruminative thoughts before and after the interventions.

**Results:**

Following interventions in both studies, perceived stress and ruminative thoughts significantly declined irrespective of the kind of prompt and intervention (study 1—perceived stress: b=–0.12; *P*<.001 and ruminative thoughts: b=–0.11; *P*<.001 and study 2—perceived stress: b=–0.07; *P*=.01 and ruminative thoughts: b=–0.10; *P*=.002). AddHRVr-triggered prompts resulted in a stronger increase in HRV during the slow-paced breathing (b=0.08; *P*=.02) and mindful breathing interventions (b=0.10; *P*=.03), and elevated HRV metrics in a time frame of 10 minutes following the interventions in contrast to random prompts (study 1: b=0.12; *P*<.001 and study 2: b=0.10; *P*=.03).

**Conclusions:**

Both studies show, for the first time, that transient, nonmetabolic reductions in HRV (AddHRVr) can be used to trigger brief JITAIs in real time by wearables to stabilize autonomic function, thus potentially promoting cardiac health. The findings suggest that although psychological benefits emerged independent of the cardiac autonomic state, slow-paced breathing or directing attention for 1 minute to either the own body or nonliving objects seemed to boost autonomic flexibility when HRV was compromised.

**Trial Registration:**

German Clinical Trial Register DRKS00035684; https://www.drks.de/search/de/trial/DRKS00035684 and DRKS00035685; https://www.drks.de/search/de/trial/DRKS00035685

## Introduction

### Background

Life is not always sunny, and we regularly experience various negative feelings such as stress, anxiety, and ruminative thoughts. These subtle yet accumulative feelings and thoughts could significantly affect long-term well-being and health outcomes [1,2]. Thus, unobtrusively detecting such episodes in daily life and applying low-threshold brief interventions in the moment of highest need (ie, just-in-time adaptive interventions [JITAIs]) [3-5] could prove a highly effective treatment and prevention strategy for an increasingly stressed population. Contrary to so-called pull interventions necessitating an individual’s motivation to request an intervention when needed, push interventions aim to automatically identify moments of highest need (eg, via wearables) and deliver the appropriate intervention [6]. Thus, we aimed to validate JITAIs designed to reduce stress and perseverative cognition (ruminative thoughts) as well as autonomic dysregulation by leveraging transient nonmetabolic decreases in cardiac vagal regulation (so-called additional heart rate variability reductions [AddHRVr]) [7,8].

Heart rate variability (HRV), and particularly the root mean square of successive differences (RMSSDs) metric of HRV, is considered to reflect vagally mediated HRV (vmHRV) and could thus constitute a particularly sensitive tool to identify such episodes of psychophysiological vulnerability, because it signifies a complex interplay between the autonomic nervous system and the central nervous system [9-11]. Specifically, the vagus nerve, as the primary parasympathetic nerve and major constituent of HRV, ensures rapid communication between the brain and the heart (approximately 200 ms), with afferent fibers (from the heart to the brain) outweighing efferent fibers (from the brain to the heart). Hence, vmHRV could signal cognitive function, emotion regulation, and states of stress and psychophysiological vulnerability, among others [10,12,13]. Thus, analyzing the rhythm of the heart throughout everyday life situations may inform about the beneficial or compromised psychosocial functioning of an organism in an ever-changing environment. Without the need for excessive, time-consuming, and burdensome self-reports of perceived stress, letting autonomic regulation decide about the right moment of potential need, the studies in this report aimed for a feasible yet efficient tool to inform JITAIs. Hence, nonmetabolic HRV reductions (ie, AddHRVr) were used to trigger JITAIs, addressing both physiological and psychological indices of well-being.

Recently, we proposed a simulation-based approach to determine individualized trigger settings for AddHRVr and assess their sensitivity for psychosocial states [14]. This approach is based on RMSSD as a measure of vagally sensitive HRV and involves 2 steps (for an overview, please refer to Multimedia Appendix 1 [15]). First, a calibration period aims to derive personalized regression parameters for the algorithm. Specifically, using 1-minute segments of data across a day (12 h), RMSSD is regressed on bodily movement for each individual. The algorithm then uses individualized RMSSD thresholds (>0.5 SD) to identify segments with substantial RMSSD reduction compared to predicted values derived from the calibration. Such AddHRVr (ie, nonmetabolic) [7,8] are tracked in the following days, and a trigger is emitted when a predefined number of AddHRVr occur within a specific time interval [15]. Because AddHRVr is a metric provided every minute, and short-term reductions in RMSSD independent of bodily movement could signal diverse psychosocial states, including positive affect [16], the exact number of segments in a predefined time window is crucial for the validity of an AddHRVr trigger. Notably, in our previous simulation study, varying the analysis window from 2 to 30 minutes and the corresponding threshold from 1 to 29 minutes, a “13 out of 28” algorithm had a power of 0.806 to detect episodes of low social quality interactions, thus suggesting a good performance of this trigger setting [14]. Precisely, when 13 one-minute segments of RMSSD in a time window of roughly half an hour (28 min) were at least 0.5 SD lower than the predicted, given a certain amount of bodily movement (signaling AddHRVr), adverse psychosocial feeling states could be predicted with good power. Hence, we used this algorithm setting (“13 out of 28”) to trigger JITAIs in 2 independent studies. We decided on 2 independent studies to reduce the complexity of the design and allow replication in 2 independent samples.

### Study 1: Slow-Paced Breathing at 0.1 Hz

In study 1, we aimed to use an easy-to-implement brief intervention, which most people can perform quite unobtrusively in various contexts in everyday life. Specifically, slow-paced breathing (0.1 Hz; approximately 6 breaths per min) has been suggested as a powerful and easy-to-learn technique to improve both mental and physical functions [17-21]. In particular, 0.1 Hz breathing has a direct effect on autonomic regulation via strengthening vagal efference and stimulating the baroreceptor reflex, which also positively affects higher central nervous system functioning [10,20]. In previous research, we evaluated acute and chronic psychophysiological effects of slow-paced breathing interventions and demonstrated beneficial effects on both psychological and physiological function [10,22-24]. Other research [20,25] reported a reduction in anxiety and perceived stress following 0.1 Hz breathing, although experimental research could not confirm significant effects for acute stress reactivity [26]. Beneficial effects of slow-paced breathing have also been reported for individuals with hypertension and cardiovascular disorders, thus confirming its clinical usefulness [27,28]. Taken together, slow-paced breathing at 0.1 Hz could benefit both autonomic regulation and psychological health. It is easy to learn and can be applied unobtrusively in various daily life contexts (eg, during work, leisure time, or travel) via smartphones [29,30].

### Study 2: Mindful Breathing

Of note, previous research demonstrated that brief mindfulness (breathing) exercises might be effective in increasing HRV [31-34] and improving well-being via reducing distress and rumination [35,36]. Mindful breathing refers to a technique that aims to facilitate staying in contact with (ie, directing attention to) one’s own breath in a nonjudgmental way. Notably, the design of study 2 allowed it to extend study 1 by including an active control condition. Thus, we could compare the effects of brief mindfulness breathing exercises with external attention exercises, both following either an AddHRVr or a random trigger. We predicted that mindful breathing exercises, when triggered by AddHRVr, are more effective than external focus interventions with respect to perceived stress, ruminative thoughts, and HRV.

### Aims and Hypotheses

In study 1, we aimed to examine whether a 0.1-Hz breathing JITAI would be particularly beneficial when autonomic function is compromised as indexed by AddHRVr. We used a microrandomized trial to compare the effectiveness of AddHRVr-triggered and randomly triggered interventions. Precisely, participants in this preregistered study [37] performed breathing exercises of 1 minute contingent on a prompt on their smartphone. We hypothesized that perceived stress and ruminative thoughts would diminish more strongly following AddHRVr (vs random)–triggered interventions and that HRV (specifically, RMSSD) would increase more strongly during and following the AddHRVr-triggered slow-paced breathing intervention as compared to random prompts.

Likewise, in study 2, we aimed to examine whether brief attention focus exercises are similarly useful for reducing perceived stress, ruminative thoughts, and cardiac autonomic regulation. Specifically, we used a microrandomized design to compare the effects of an internal (respiration) focused mindfulness intervention and an external focus intervention, each triggered either by AddHRVr or randomly. We hypothesized that mindful breathing exercises would decrease perceived stress and rumination and increase HRV relative to an external attention exercise.

## Methods

### Participants

#### Study 1

Overall, 69 individuals participated in the ecological momentary assessment for 3 consecutive days, following a 12-h calibration period. Data loss due to excessive electrocardiographic artifacts (n=7) or positive slopes during the calibration period (n=2) resulted in a final sample of 60 participants (n=45, 75% women, n=15, 25% men, and n=0 diverse, nonbinary, or other). They had a mean age of 22.88 (SD 4.56) years and a mean BMI of 21.83 (SD 3.30) kg/m^2^. Most of the participants were students (48/60, 80%) and nonsmokers (45/60, 75%), and they reported regular physical exercise (30/60, 50%). Information about race, ethnicity, or socioeconomic status was not gathered. Inclusion criteria were ages between 18 and 45 years, no self-reported cardiovascular or mental disorders, and no self-reported psychotropic or cardiovascular medication. A power analysis was conducted a priori based on simulation studies [38], focusing on direct within-person effects of small to medium size (assuming a medium-sized interclass coefficient and approximately 30 prompts per participant). The resulting sample size was 60 participants.

#### Study 2

A power analysis for microrandomized trials was conducted using the Micro-Randomized Trials–Sample Size tool [39,40]. A time frame of 4 days was applied with a power of 0.80 and a treatment effect of 0.2 with a randomization probability of 0.40 and the level of significance fixed at *P*<.05. The expected availability was set to 0.75, and the proximal treatment effect was set to constant. This resulted in a recommended sample size of 30. Decreasing the treatment effect to 0.15 resulted in a sample size of 51. Hence, we strived for a sample size of 50 individuals to detect smaller-sized effects. The final sample comprised 51 participants, of whom 39 (76%) were women and 12 (24%) were men. The same inclusion and exclusion criteria were applied as in study 1. There were no gender-diverse individuals in this sample. The mean age of the participants was 23.33 (SD 3.94) years, with most of them being students (39/51, 76%). There were 20% (10/51) smokers, and 65% (33/51) of the participants reported regular physical activity. Mean BMI was 23.20 (SD 4.60) kg/m^2^. Information about race, ethnicity, or socioeconomic status was not gathered. One participant was excluded due to implausible positive slopes between RMSSD and bodily movement during calibration, resulting in a final sample size of 50.

### Ethical Considerations

Both studies were approved by the local ethics committee (study 1: GZ. 39/93/63 ex 2022/23 and study 2: GZ. 39/54/63/ ex 2023/24) and preregistered via Open Science Framework (study 1 [37] and study 2 [41]). Written informed consent was obtained for both studies, and trials were registered at the German Clinical Trials Register (study 1: DRKS00035684 and study 2: DRKS00035685). Participants were explicitly informed about the psychological concepts of the studies, study duration, and the concepts of JITAIs, as well as the interventions applied. They agreed that data would be analyzed in summary form and used for scientific purposes only (including publication). They were informed that they could withdraw participation at any time without giving reasons and could request deletion of their data upon request. They were also informed to get information in case ectopic beats or arrhythmias were detected to allow clarification by a cardiologist. Data were pseudoanonymized via a personal code not attached to names or other identifying information. Participants received no financial compensation for participation but could receive course credit if applicable.

### Study Design

#### Study 1

**Figure 1:**
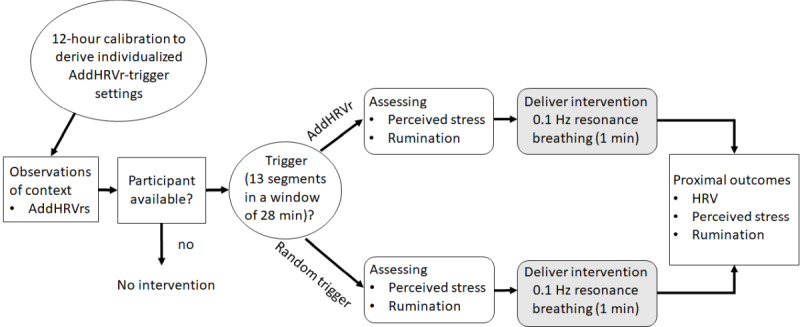
Schematic illustration of the procedure of study 1. A slow-paced breathing intervention (0.1 Hz) was either triggered at random points in time or contingent on additional (ie, nonmetabolic) heart rate variability reductions (AddHRVr).

The study followed a microrandomized design [42,43], triggering a brief intervention either by episodes of psychophysiological vulnerability (AddHRVr trigger) or at random times. The design is outlined in Figure 1.

Using a calibration period of 12 hours, we derived the regression parameters slope and intercept for each individual (refer to Multimedia Appendix 2 for descriptive statistics) and fed these parameters into the real-time working algorithm for the subsequent days. On the basis of these personalized regression parameters, the intervention was triggered either when RMSSD was 0.5 SD less than the predicted value in 13 minutes during a time frame of 28 minutes (AddHRVr trigger) or at random times (Multimedia Appendix 1). Before each intervention and immediately thereafter, participants provided ratings of perceived stress and ruminative thoughts, and HRV (ie, RMSSD) was recorded 10 minutes before the intervention, during the intervention, and 10 minutes thereafter. There were no restrictions for AddHRVr triggers, except a silence mode setting of 60 minutes, meaning that following an AddHRVr trigger, there was a period of 60 minutes without any emitted alarm. Data collection took place on 4 consecutive days (including the calibration period).

#### Study 2

**Figure 2:**
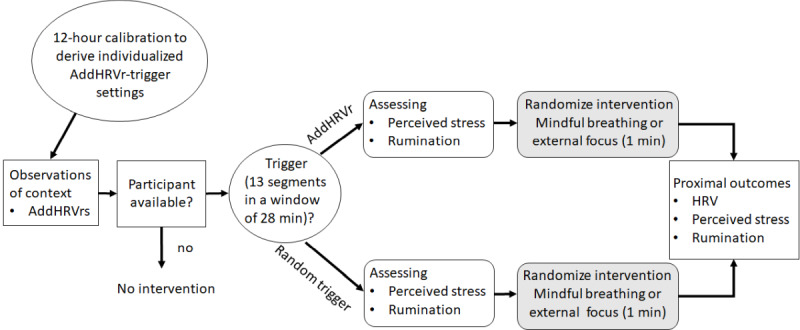
Schematic illustration of the procedure of study 2. Two interventions requiring attention either to internal or external cues were either triggered at random points in time or contingent on additional (ie, nonmetabolic) heart rate variability reductions (AddHRVr).

The study design was comparable to study 1; however, an active control condition (randomized within individuals) was implemented in addition to mindful breathing exercises (Figure 2).

### Variables and Instruments

#### Perceived Stress

Perceived stress was assessed via 4 items adapted from the Perceived Stress Scale [44,45]. The following items were rated on a 5-point Likert scale between 1 (not at all) and 5 (very much): “Do you feel that you can cope with things?” (reverse coded), “Do you feel that you’re on top of things?” (reverse coded), “Do you feel you have control over things?” (reverse coded), and “Do you feel nervous or stressed?” Items were aggregated for each prompt to yield a mean score of perceived stress. To evaluate the reliability of this measure, generalizability theory analysis [46] was applied via the package *psych* (version 2.4.3 [47]) in R (version 4.4.0 [48]). Generalizability theory analysis allows us to distinguish the reliability of the measure in reliability between persons (comparable to traditional measures of internal consistency) and within persons. For study 1, between-person reliability was excellent (before intervention: *R_KRn_*=0.98 and afterintervention: *R_KRn_*=0.99), documenting that differences in stress ratings between individuals could be assessed with high reliability. Moreover, within-person reliability (before intervention: *R_Cn_*=0.68 and afterintervention: *R_Cn_*=0.69) seemed satisfactory, thus indicating that the assessment of change within each person was reliable. The mean average score across individuals and occasions was 2.23 (SD 0.79) before assessment and 2.11 (SD 0.76) after assessment, with an intraindividual range from 1 to 5.

Likewise, for study 2, the scale showed excellent between-person reliability (before intervention: *R_KRn_*=0.97 and after intervention: *R_KRn_*=0.98) and satisfactory within-person reliability (before intervention: *R_Cn_*=0.62 and after intervention: *R_Cn_*=0.65). The mean average score across individuals and occasions was 1.91 (SD 0.84) for before assessment and 1.84 (SD 0.79) for after assessment, with an intraindividual range from 1 to 5. Stress scores were lower as compared to study 1.

#### Rumination

In study 1, ruminative thoughts were assessed via a single-item measure. In particular, following previous research [49], ruminative thoughts were defined as “When you worry or are ruminating about something over a period of time. It is a summary term for processes such as worrying, brooding, stuck on something, annoyed or grumbling about a problem, or brooding angrily, etc. So, it is a chain of negative thoughts that is difficult to let go of.” Upon every prompt, this introduction was presented, followed by the question “Does this apply to you at the moment?” Answers are given on a 5-point Likert scale (from 1=not at all to 5=very strongly). The mean average score across individuals was 2.14 (SD 1.02) before assessment and 2.02 (SD 0.97) after assessment, with an intraindividual range between 1 and 5.

In study 2, ruminative thoughts were assessed with a slightly adjusted item from study 1, as published by Du et al [50] “I’m recalling all my shortcomings, failures and the things that I did wrong.” We decided on this alternative assessment instead of the rather long introductory text applied in study 1 for time-economic reasons. Answers were given on a 5-point Likert scale between the poles 1 (“does not apply”) and 5 (“does fully apply”). The mean average score across individuals was 1.78 (SD 1.03) before assessment, and 1.68 (SD 0.94) after assessment with an intraindividual range between 1 and 5. Hence, rumination scores were lower than in study 1.

#### HRV Measurement

We applied the EcgMove4 device (movisens GmbH) to record the electrocardiogram (with 1024 Hz), bodily movement and body position (by a 3D accelerosensor 64 Hz), and air pressure (8 Hz) for 4 weekdays (9 AM-9 PM; except nighttime). The first day (12 hours on Monday) served as a calibration day to determine the individual regression parameters between RMSSD and bodily movement for each individual (intercept, slope, and SD of RMSSD; Multimedia Appendix 2). Validation of RMSSD (analyzed for each minute) was verified by an in-built algorithm. In case of severe artifacts, the 1-minute segment was set to missing. Individuals exhibiting positive slopes were excluded from analysis (1/51, 0.2%). The regression parameters were then used to detect AddHRVr and emit a trigger in the subsequent 3 days (from Wednesday to Friday [15]) to initiate smartphone prompts. As previously outlined, the trigger was emitted when 13 one-minute segments of RMSSD in a time window of roughly half an hour (28 min) were at least 0.5 SD lower than the predicted, given a certain amount of bodily movement (Multimedia Appendix 1). AddHRVr triggers were complemented by random triggers. For AddHRVr triggers, we applied a silent setting of 60 minutes, meaning that 1 AddHRVr trigger was emitted per hour at maximum.

#### Metabolic Equivalents

Metabolic equivalents (METs) were derived from the EcgMove4 sensor, which records acceleration (in g) via a triaxial accelerometer and air pressure. The movisens DataAnalyser software calculates the METs in 2 steps. First, the activity class is estimated based on acceleration and barometric signals. Second, based on the detected activity class (ie, lying, sitting, standing, and walking), the algorithm chooses the corresponding model for MET estimations, which takes the acceleration metric, the barometric data, and personal parameters (ie, age, gender, weight, and height) into account.

### Overview of JITAIs

#### Study 1: Slow-Paced Breathing Intervention (0.1 Hz)

**Figure 3:**
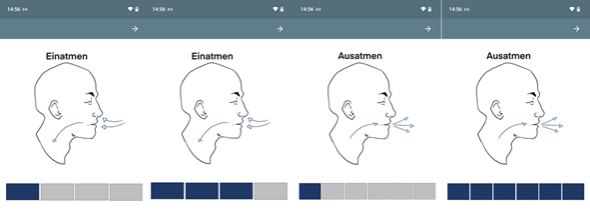
App-based 0.1 Hz breathing intervention. Inhalation (“Einatmen”) was paced for 4 seconds and exhalation (“Ausatmen”) for 6 seconds, as guided by a progress bar.

Following each ecological momentary assessment (random and AddHRVr-triggered), participants were instructed to breathe slowly for 1 minute via a guided app (Figure 3). Inhalation was set to 4 seconds and exhalation to 6 seconds, thus arriving at a breathing frequency of 6 breaths per minute (0.1 Hz) [10,20,24]. For exploratory purposes, the breathing intervention was partially complemented by hand muscle contractions during inhalation and relaxation during exhalation. However, further analysis confirmed that both intervention types did not result in different HRV patterns (data available upon request); thus, we abstained from controlling hand contractions in analyses. Exact instructions can be found in Multimedia Appendix 3.

#### Study 2: Mindful Breathing and External Cues Intervention

**Figure 4:**
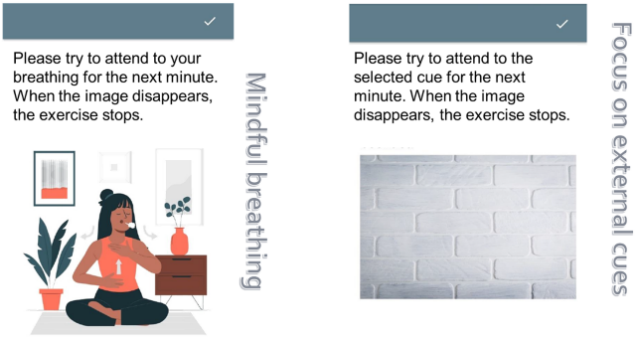
Images of the app-based interventions showing brief instructions, which were delivered following either additional heart rate variability reduction triggers or random triggers. The mindful breathing intervention (left side) instructed participants to direct their attention toward mindful breathing, while the external attention task (right side) instructed them to focus their attention on a neutral object in the environment. Interventions were performed for 1 minute and randomized for trigger type.

We applied brief, unobtrusive cognitive exercises (1 min length), which aimed to direct attention either to the own respiration (mindful breathing) or to a neutral (nonliving) object in the outside world. Interventions were presented in random order for each participant. The mindful breathing intervention was informed by research documenting reduced reactivity to repetitive thoughts [51] and calming effects on cardiac activity [31-33] resulting from mindful breathing. Regarding the external focus exercises, participants were instructed to focus on a neutral, nonliving object in their surroundings. Interventions are visualized in Figure 4. Detailed instructions are provided in Multimedia Appendix 3.

### Preregistration and Deviation Thereof

#### Study 1

Some deviations from the preregistered protocol need to be outlined. First, we specified to assess weight-to-height ratio in addition to BMI. For reasons of time economy, we focused on BMI and are confident that we have captured tendencies of overweight reasonably well. Second, we specified to control for smoking, age, gender, and BMI. However, because all these covariates turned out to be nonsignificant and to avoid overspecification of the model, we did not include all these variables in the final model but focused on concurrent smoking and METs when analyzing HRV variables. Third, we did not specify the HRV measure as the outcome variable in the preregistration. However, as outlined in previous research, RMSSD is considered a core measure of this approach [7,8,14]. Nevertheless, to evaluate a broader spectrum of HRV trajectories before and following the intervention, as recommended by some authors [52], we additionally analyzed the SD of normal-to-normal (SDNN) interbeat intervals, which is considered an indicator of autonomic regulation of HRV [52], and the vagally sensitive high-frequency HRV (HF-HRV; 0.15-0.40 Hz) to avoid selective reporting of HRV measures.

#### Study 2

We preregistered 5 hypotheses in this preregistration [41]. To align this research with study 1, we concentrated on three of them: (1) a mindfulness-based breathing intervention leads to a greater increase in HRV after a detected, nonmetabolic HRV reduction than a control intervention; (2) a mindfulness-based breathing intervention leads to a greater decrease in subjective stress after a detected, nonmetabolic HRV reduction than a control intervention; and (3) a mindfulness-based breathing intervention leads to a greater decrease in rumination after a detected, nonmetabolic HRV reduction than a control intervention.

### Data Parametrization and Statistical Analysis

In study 1, participants received, on average, 45 (SD 8.38; range 31-65) prompts (total n=2571), of which 1782 (69.31%) were answered, 99 (3.85%) were dismissed, 477 (18.55%) were ignored, and 194 (7.54%) were incomplete. Notably, 33% (20/60) of the participants received AddHRVr triggers, while 67% (40/60) did not. On average, 4.14 (SD 7.10) AddHRVr triggers were emitted with an interindividual range between 0 and 26. In study 2, participants received, on average, 43 (SD 11.97; range 24-71; total n=2174) prompts, of which 1307 (60.12%) were answered, 163 (7.49%) were dismissed, 626 (28.79%) were ignored, and 29 (1.33%) were incomplete. Of note, 12% (6/50) of the participants received no AddHRVr triggers, while the remaining participants (44/50, 88%) received between 1 and 39 AddHRVr triggers. On average, 14.41 (SD 10.72) AddHRVr triggers were emitted per individual, which was considerably more than in study 1.

To evaluate the effectiveness of the JITAIs, we applied multilevel (mixed effects) models predicting perceived stress, rumination, and RMSSD, respectively, by AddHRVr trigger (vs random trigger) and time (before intervention vs intervention and before intervention vs after intervention) and the interaction of these variables. For study 2, intervention type (mindful breathing vs external focus) was additionally added to the models. We used RMSSD as the core outcome variable of HRV because it has been particularly recommended in previous ambulatory research [53] and represents the most often studied ambulatory marker of vmHRV [54-56]. RMSSD was quantified as the mean of the 10 minutes before each prompt. Importantly, we controlled for METs as a covariate (mean of 10 min), because the breathing intervention could, in principle, be performed in any body position and independent of bodily movement (eg, during walking or standing or sitting still). RMSSD was analyzed in a time frame of 10 minutes before the intervention (coded as –1), during the intervention (coded as 0), and the following 10 minutes (coded as 1). Of note, to evaluate effects during the interventions, we applied a time frame of SD 2 minutes to account for potential inaccuracy of timing between smartphones and EcgMove4 devices.

To evaluate the robustness of intervention effects on HRV, we also analyzed HF-HRV and SDNN [52]. Specifically, HF-HRV is considered a frequency-domain indicator of vagally sensitive HRV, and SDNN indicates overall autonomic regulation of cardiac activity. To compare adherence with the 0.1 Hz breathing interventions during both types of triggers (AddHRVr vs random) in study 1, we also analyzed low-frequency HRV (LF-HRV; 0.04-0.15 Hz) during the interventions. It should be noted in this respect that respiratory sinus arrhythmia (ie, the fluctuation of heart rate concordant with breathing) shifts to the LF-band (0.04-0.15 Hz) during breathing with 6 breaths per minute (corresponding to 0.1 Hz) [20]. HRV measures were logarithmized (ln) before analysis to account for skewness.

The interaction between prompt (random vs AddHRVr) and time (before intervention vs intervention and before intervention vs after intervention) would indicate the effectiveness of the AddHRVr-triggered intervention on the outcome variables mentioned earlier. For analyzing study 2, we also centered intervention type within persons. Throughout the analyses, we additionally controlled for age, gender, BMI, METs, and momentary smoking. Since age, gender, and BMI did not change any of the associations of interest, they were removed from the final models. Continuous predictor variables were centered. Moreover, we centered trigger and intervention (study 2) on the within-person level to allow interpretation of within-person effects [57].

Analyses were conducted using R (version 4.4.0 [48]) with the package *lme4* (version 1.1-35.3 [58]). Note that b refers to the unstandardized estimate of the relationships. We fixed the level of significance at *P*<.05 (2-tailed).

## Results

### Study 1

#### Overview

In the first step, we aimed to evaluate whether the slow-paced breathing interventions were performed equally for both trigger conditions. Therefore, we analyzed LF-HRV before and during the breathing exercises via multilevel modeling. Precisely, we predicted lnLF-HRV by the interaction of time (10 min before the intervention coded as –1 vs 2 min around the intervention coded as 0) and trigger (AddHRVr trigger vs random trigger). Momentary smoking and METs served as covariates. The interaction was not significant (b=0.06; *P*=.32), thus suggesting that the slow-paced breathing exercises were not performed differently between the 2 types of triggers (AddHRVr trigger vs random trigger).

#### Perceived Stress and Rumination

Next, we analyzed a model predicting both perceived stress and ruminative thoughts by trigger type (AddHRVr vs random) and time (before vs after intervention). It turned out that both variables significantly declined from pre- to postintervention (perceived stress: b=–0.12; *P*<.001 and ruminative thoughts: b=–0.11; *P*<.001), but there were no significant interactions between trigger and time, neither for perceived stress (b=0.02; *P*=.82) nor for rumination (b=–0.06; *P*=.54).

#### HRV Measurement

First, we analyzed whether RMSSD differed in the course of the intervention between trigger conditions. Therefore, lnRMSSD was averaged in a time window of 10 minutes before the intervention, around the 2 minutes of intervention, and 10 minutes thereafter. METs and concurrent smoking were treated as covariates in this model to account for metabolic adjustments. Findings are depicted in Tables 1 and 2.

**Table 1:** Multilevel regression predicting logarithmized root mean square of successive differences (lnRMSSD) before, during, and after the slow-paced breathing intervention (N=60).

Predictors	lnRMSSD	
	Estimate, b (95% CI)	*P* value
Intercept	3.53 (3.44 to 3.62)	<.001
Metabolic equivalents	–0.35 (–0.36 to –0.35)	<.001
Smoking (no vs yes)	–0.03 (–0.06 to –0.00)	.03
Trigger (0=random vs 1=AddHRVr^a^)	–0.35 –0.38 to –0.32	<.001
Time (before intervention vs during intervention [breathing])	0.17 (0.16 to 0.19)	<.001
Time (before intervention vs after intervention)	0.03 (0.02 to 0.04)	<.001
Trigger×time (before intervention vs during intervention [breathing])	0.08 (0.02 to 0.14)	.02
Trigger×time (before intervention vs after intervention)	0.12 (0.07 to 0.16)	<.001

^a^AddHRVr: additional heart rate variability reductions.

**Table 2:** Random effects estimates (N=60).

Parameters	Values
Within-group variance (σ^2^)	0.23
Between-group variance (τ_00_ [participant])	0.13
Interclass correlation coefficient	0.35
Observations, n	33,758
Marginal *R*^2^	0.162
Conditional *R*^2^	0.457

**Figure 5:**
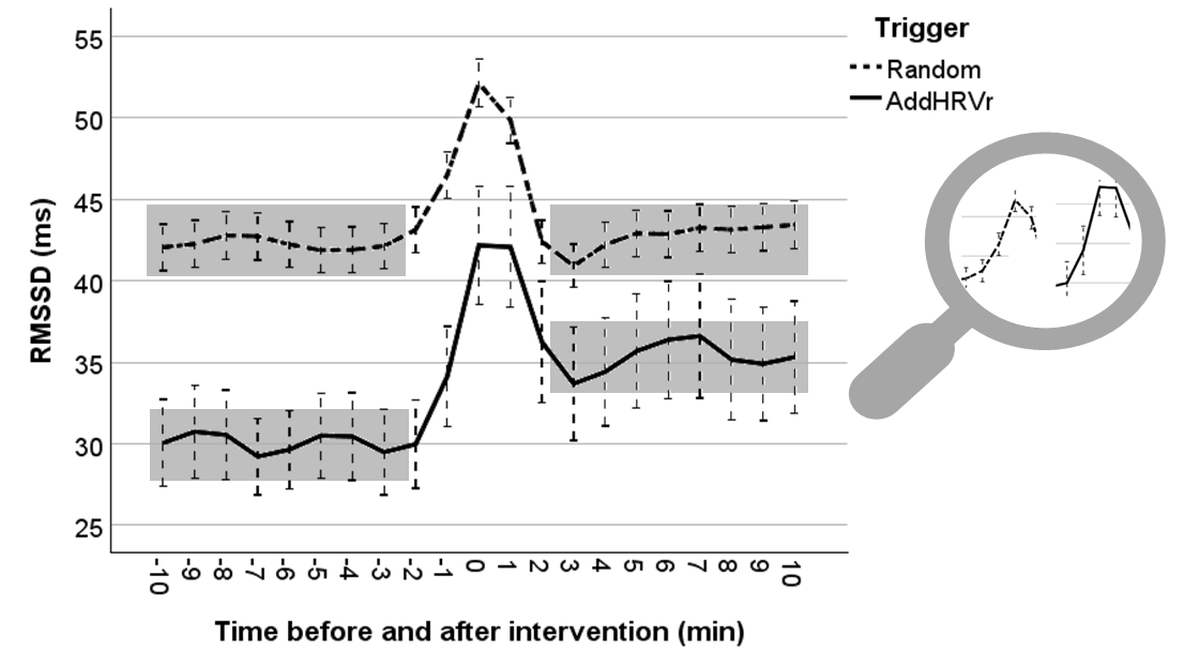
Trajectories of vagally mediated heart rate variability (root mean square of successive difference [RMSSD]) 10 minutes before and after the prompt, respectively. The upper stippled line represents RMSSD following random prompts, and the lower solid line indicates RMSSD following an additional heart rate variability reductions (AddHRVr) trigger. Stippled whiskers indicate 95% CIs. The shadowed areas visualize the significant interaction between the trigger and the prepost assessment, and the stronger increase in RMSSD to the AddHRVr-triggered intervention is magnified on the right side.

According to expectations, the increase in lnRMSSD during the intervention was significantly stronger following AddHRVr triggers as compared to random triggers (trigger by time [before intervention vs intervention]: b=0.08; *P*=.02). Most importantly, lnRMSSD remained significantly higher 10 minutes after the intervention in the AddHRVr-trigger condition as compared to the random trigger condition (trigger by time [before intervention vs after intervention]: b=0.12; *P*<.001). Furthermore, increasing METs and concurrent smoking were accompanied by significantly lower lnRMSSD (main effect MET: b=–0.35; *P*<.001 and main effect smoking: b=–0.03; *P*=.03). The RMSSD trajectories for both trigger conditions are visualized in Figure 5 (note that we used unadjusted raw scores here to allow for evaluating the plausibility of the data).

#### Sensitivity Analysis

To evaluate the sensitivity of the HRV-increasing effect of the slow-paced breathing intervention, we analyzed alternative indicators of HRV (HF-HRV and SDNN). Notably, the HRV-enhancing effect of the slow breathing intervention to an AddHRVr trigger relative to a random prompt was also significant for lnHF-HRV (trigger by time [before intervention vs intervention]: b=0.16; *P*=.01 and trigger by time [before intervention vs after intervention]: *b*=0.21; *P*<.001) and partially significant for lnSDNN (trigger by time [before intervention vs intervention]: b=0.05; *P*=.07 and trigger by time [before intervention vs after intervention]: b=0.06; *P*<.001), thus suggesting general effectiveness of the JITAI on HRV.

#### Evaluating Spontaneous Recovery of HRV

In the final step, we aimed to evaluate whether the increase of HRV (in particular, RMSSD) following the intervention is specific to the intervention or merely reflects a spontaneous rebound effect. Of note, when nonmetabolic HRV is lower during a longer period, an increase to normal levels, irrespective of any intervention, could be expected. Hence, we scanned the dataset offline for potential AddHRVr triggers that were not evoked (eg, because the silence period prevented an additional prompt). In the following discussion, we refer to these triggers as virtual AddHRVr triggers because they show the same characteristics as the AddHRVr trigger (13 segments in a time window of 28 min) but had never been evoked in real time. We then calculated the interaction between trigger type (virtual AddHRVr triggers vs random triggers vs regular AddHRVr triggers) and time (10 min before intervention vs 10 minutes after intervention). The interaction effect trigger (virtual vs regular AddHRVr) by time (before intervention vs after intervention) was highly significant (b=0.08; *P*<.001; Multimedia Appendix 4), indicating that RMSSD increased more strongly following AddHRVr triggers with the intervention (relative to before intervention) than following virtual AddHRVr triggers.

### Study 2

#### Perceived Stress and Rumination

Similar to study 1, we found significant effects of time (before intervention vs after intervention) for both perceived stress (b=–0.07; *P*=.01) and rumination (b=–0.10; *P*=.002), thus documenting significant decreases in both variables from before intervention to after intervention. No other effects were significant. Specifically, there were no significant interactions between trigger type, intervention type, and time for perceived stress (b=–0.03; *P*=.81) and rumination (b=–0.18; *P*=.18).

#### HRV Measurement

**Figure 6:**
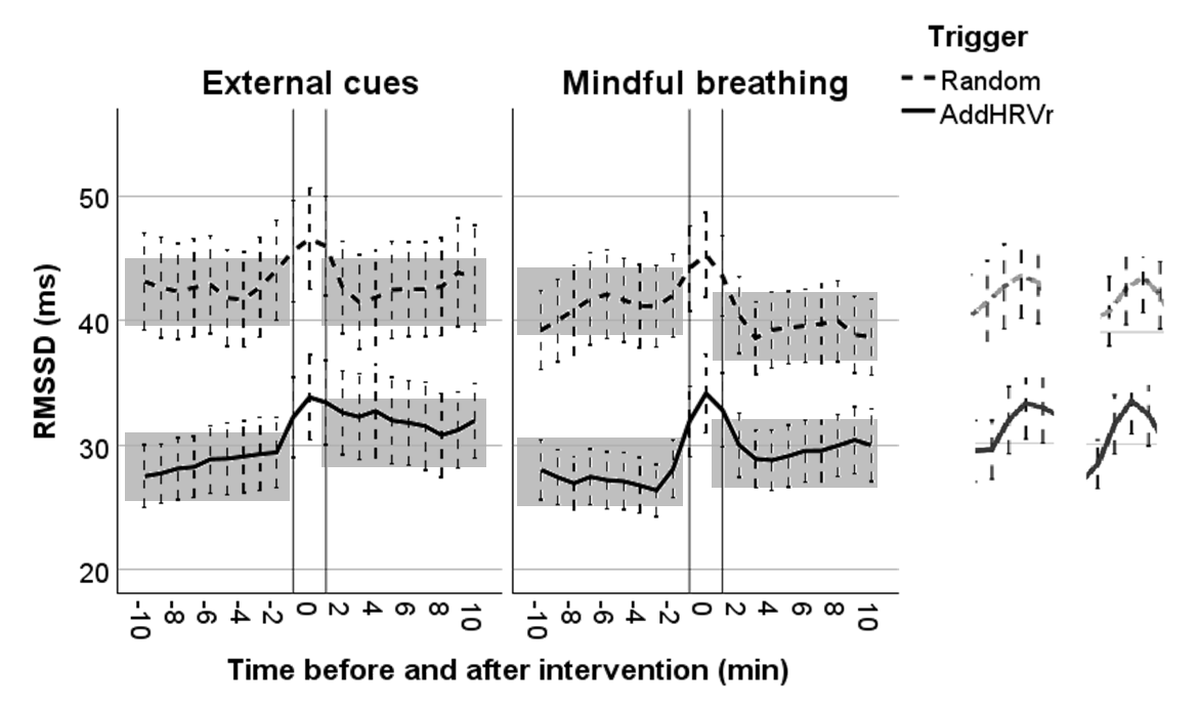
Trajectories of vagally mediated heart rate variability (root mean square of successive difference [RMSSD]) 10 minutes before and after the prompt, respectively. The upper stippled line represents RMSSD following random prompts, and the lower solid line indicates RMSSD following an additional heart rate variability reductions (AddHRVr) trigger. The external attention intervention (external cues) is depicted on the left side, and the mindful breathing intervention on the right side. Stippled whiskers indicate 95% CIs. The shadowed areas visualize the significant interaction between the trigger and the prepost assessment, and the stronger increases in RMSSD to the AddHRVr-triggered interventions are magnified on the right side.

Analysis of lnRMSSD revealed a significant 3-way interaction between trigger, intervention type, and time (before intervention vs intervention phase: b=0.10; *P*=.03), thus suggesting that the change from before intervention to intervention phase differed as a function of trigger and intervention type (Tables 3 and 4). To further qualify this interaction, we rescaled both trigger and intervention type to examine the interaction of the remaining variables. When the external cues intervention type was set to reference, the interaction between trigger and time (before intervention vs intervention) was not significant (b=–0.007; *P*=.83), thus suggesting that for the external cues condition, the increase in RMSSD from before intervention to the intervention phase was not dependent on the type of trigger (left column in Figure 6). However, for the mindful breathing intervention, this interaction proved significant (b=0.09; *P*=.004), thus indicating that when performed following an AddHRVr trigger, mindful breathing resulted in a significantly larger increase in RMSSD as compared to following a random trigger. This finding is illustrated in Figure 6 (right column). Further analyses indicated that when the random trigger was set to reference, the interaction of intervention type and time (before intervention vs intervention) was not significant (b=–0.01; *P*=.63; top lines left and right in Figure 6), thus indicating that following random triggers, both types of interventions resulted in roughly the same increase in RMSSD. Of note, when the AddHRVr trigger was set to reference, the interaction was significant (b=0.07; *P*=.01), thus documenting that when triggered by AddHRVr, the mindful breathing intervention resulted in significantly larger RMSSD increases as compared to the external attention intervention (bottom lines left and right in Figure 6).

**Table 3:** Multilevel regression predicting logarithmized root mean square of successive differences (lnRMSSD) before, during, and after intervention for both types of triggers (N=50).

Predictors	lnRMSSD	
	Estimate, b (95% CI)	*P* value
Intercept	3.41 (3.29 to 3.53)	<.001
Metabolic equivalents	–0.22 (–0.23 to –0.21)	<.001
Smoking (no vs yes)	–0.28 (–0.31 to –0.25)	<.001
Trigger (0=random vs 1=AddHRVr^a^)	–0.35 (–0.37 to –0.33)	<.001
Intervention (mindful breathing vs external focus)	–0.02 (–0.04 to 0.00)	.047
Time (before intervention vs during intervention)	0.08 (0.07 to 0.10)	<.001
Time (before intervention vs after intervention)	0.04 (0.03 to 0.06)	<.001
Trigger×intervention	–0.07 (–0.11 to –0.02)	.003
Trigger×time (before intervention vs during intervention)	0.04 (–0.00 to 0.08)	.05
Trigger×time (before intervention vs after intervention)	0.09 (0.06 to 0.12)	<.001
Intervention×time (before intervention vs during intervention)	0.03 (–0.01 to 0.07)	.10
Intervention×time (before intervention vs after intervention)	–0.00 (–0.03 to 0.03)	.93
Trigger×intervention×time (before intervention vs during intervention)	0.10 (0.01 to 0.18)	.03
Trigger×intervention×time (before intervention vs after intervention)	0.04 (–0.02 to 0.10)	.23

^a^AddHRVr: additional heart rate variability reductions.

**Table 4:** Random effects estimates (N=50).

Parameters	Values
Within-group variance (σ^2^)	0.21
Between-group variance (τ_00_ [participant])	0.19
Interclass correlation coefficient	0.47
Observations, n	20,418
Marginal *R*^2^	0.127
Conditional *R*^2^	0.534

Furthermore, the significant trigger by time (before intervention vs after intervention) interaction (b=0.09; *P*<.001), which was not further qualified by a 3-way interaction involving intervention type, suggests that following an AddHRVr trigger, RMSSD significantly increased from pre- to postintervention period as compared to when triggered randomly. Hence, any of the interventions performed following an AddHRVr trigger resulted in an increase of RMSSD in a time window of 10 minutes after intervention. Finally, it should be noted that similar to study 1, RMSSD was significantly lower with increasing METs (b=–0.22; *P*<.001) and when the participants smoked (b=–0.28; *P*<.001).

For illustration purposes, Figure 6 depicts the trajectories of RMSSD (raw values) unadjusted for covariates for each trigger and intervention type.

#### Sensitivity Analyses

As in study 1, sensitivity analyses were conducted for both lnHF-HRV and lnSDNN. For lnHF-HRV, the 3-way interaction between trigger, intervention type, and time (before intervention vs intervention phase) was significant (b=0.18; *P*=.049). The same was evident for lnSDNN (b=0.08; *P*=.02), thus replicating the findings for RMSSD and confirming the stronger HRV increase to the AddHRVr-triggered mindful breathing interventions. Furthermore, the 2-way interaction between trigger and time (before intervention vs postintervention phase) could be confirmed for both lnHF-HRV (b=0.17; *P*<.001) and lnSDNN (b=0.05; *P*<.001), respectively. Hence, the generally facilitating recovery effects of both types of interventions appeared robust for different HRV metrics.

#### Evaluating Spontaneous Recovery of HRV

Mirroring the approach of study 1, we finally evaluated whether the increase in HRV after both interventions contingent on AddHRVr triggers was higher as compared to a spontaneous rebound effect of HRV following offline-determined virtual AddHRVr triggers (without interventions). The interaction between trigger (virtual AddHRVr trigger vs AddHRVr trigger with intervention) and time (before intervention vs after intervention) was significant (b=0.07; *P*<.001; Multimedia Appendix 5), thus documenting that AddHRVr-triggered interventions resulted in a significantly stronger increase in RMSSD as compared to virtual AddHRVr triggers without interventions. Hence, the applied interventions seemed to drive the boosting effect on HRV and not a spontaneous rebound following low HRV. Interestingly, the effect size was similar to that of study 1.

## Discussion

### Principal Findings

In both studies, we aimed to evaluate the effectiveness of physiologically triggered (ie, AddHRVr) JITAIs to restore psychological well-being and autonomic flexibility. In study 1, we used a brief (1 min) slow-paced breathing intervention, and in study 2 we applied brief mindful breathing and external focus exercises. The findings document that such 1-minute interventions resulted in a general decline of stress and rumination, independent of the right moment. Both slow-paced breathing and mindful breathing interventions applied at the moments of compromised cardiac vagal function (ie, following AddHRVr triggers) resulted in a stronger increase or renewed increase of HRV. Precisely, we observed higher HRV during the breathing interventions and up to 10 minutes thereafter when the intervention was triggered by AddHRVr as compared to randomly triggered prompts. It should be mentioned in this respect that in study 2 the renewed increase in HRV following the interventions could also be observed for the external focus exercises, thus suggesting a rather nonspecific intervention effect in reestablishing autonomic flexibility.

Importantly, AddHRVr contingent increases in HRV during and after the interventions could be verified for different indices of HRV (namely RMSSD, HF-HRV, and SDNN), thus suggesting rather broad-bended beneficial effects on cardiac autonomic regulation. Hence, triggering brief interventions in moments of low HRV could prove particularly beneficial for stabilizing cardiac autonomic regulation, thus arguing for the just-in-time intervention concept. Importantly, the cardiac vagal rebound relative to preintervention levels observed in both studies was more pronounced than could normally be expected without interventions. Specifically, analyzing responses to so-called virtual AddHRVr triggers (nonmetabolic HRV decreases that did not evoke triggers but showed the same characteristics as the AddHRVr triggers) suggested that the increase in HRV following the AddHRVr-triggered interventions was significantly more pronounced than the natural increase of HRV following virtual AddHRVr triggers, thus excluding the interpretation of spontaneous rebound effects. Thus, it seems that shifting attention to either internal or external cues or engaging in slow breathing exercises was a main driver for the increase in cardiac autonomic flexibility.

The findings of study 1 generally align with research suggesting beneficial effects of slow breathing on emotional valence, perceived stress, and anxiety in controlled settings [18,59,60]. Moreover, the finding confirms research showing that slow breathing biofeedback increased attentional control in individuals reporting higher levels of stress [61], suggesting that slow breathing exercises are particularly useful when applied during elevated (physiological) stress. Moreover, the conceptual replication in study 2 confirms research on the beneficial effects of mindful breathing for mental health [35,36,62]. However, we need to emphasize that, similar to study 1, in contrast to the HRV effects, the psychological effects for stress and rumination were evident irrespective of the cardiac autonomic state and, moreover, independent of the type of intervention. This result aligns with research suggesting, for example, beneficial psychological effects of breathing exercises irrespective of the pace [63].

Because any of the interventions applied seemed to benefit both rumination and perceived stress independent of the cardiac autonomic state, common mechanisms inherent in both interventions need to be discussed. First and foremost, it could be assumed that both interventions facilitated distraction from ongoing activities. Distraction has been considered a powerful emotion regulation strategy [62], especially when combined with acceptance [64]. Notably, there is also neurobiological evidence that distraction as an emotion regulation strategy is associated with a comparably strong decrease in amygdala activity, thus substantiating the beneficial effects on subjective well-being [65]. Within a broader perspective, attentional deployment has been considered crucial for emotion regulation, for example, to either distract oneself from negative cues or refocus on positive cues [66]. Taken together, we would assume that the psychological effectiveness of the JITAIs applied in this research was mainly driven by the deployment of attention.

### Limitations and Recommendations

The studies had some limitations that need to be acknowledged. First and foremost, they failed to show that perceived stress and rumination could be reduced more strongly when triggered by HRV. In fact, irrespective of the trigger, both stress and ruminative thoughts were ameliorated in the course of the interventions. Consequently, unlike HRV, the psychological variables do not strongly argue for a just-in-time approach. Certainly, more research is warranted to examine the exact circumstances under which participants will benefit more strongly with respect to their psychological well-being. Alternatively, refining the trigger settings to detect psychologically vulnerable periods in time with higher accuracy and adapting the interventions to the specific needs constitutes an important task for future research [67].

Second, sample sizes for both studies were rather moderate, and the general levels of perceived stress and rumination were rather low. Future research should increase both sample sizes and days of assessment and assess more heterogeneous samples with compromised well-being to uncover effects with higher sensitivity, especially the superiority of AddHRVr-triggered interventions for perceived stress and rumination.

Third, there was no passive control intervention, thus limiting the interpretation of the findings. Thus, we could not rule out the possibility that any kind of alternative activity or disruption of everyday life activities for 1 minute may benefit well-being and cardiac autonomic regulation. Accordingly, study 2 suggests that effects on psychological variables might indeed be unspecific to the intervention applied. Relatedly, we cannot rule out that the reduction of perceived stress and rumination indicates mere order effects or psychological adaptation to the repeated assessment or even socially desirable responding. Taken together, a control group without interventions or a within-person condition without intervention is needed in future research to ascribe the effects solely to the interventions.

Fourth, while we analyzed immediate and delayed cardiac autonomic effects of the interventions, it should be kept in mind that the analyzed time window for the postintervention effects was restricted to 10 minutes. It would be worthwhile to analyze longer time scales to examine more sustained effects of the 1-minute interventions. Fifth, in study 1, only about 33% (20/60) of the participants received AddHRVr triggers, thus suggesting that either the trigger setting was too conservative, ultimately missing less severe episodes of cardiac vagal reductions in most participants, or calibration was not sensitive enough to identify episodes of psychophysiological vulnerability. Notably, the same trigger settings were deemed more suitable in study 2, with 88% (44/50) of the participants receiving AddHRVr triggers. We are not sure about the cause of this discrepancy. However, it should be noted in this respect that the performance of the trigger depends on the generalizability of the 12-hour calibration period. If physical activity changes substantially after the calibration period, the algorithm settings may become inaccurate, leading to less reliable triggers. Future research is advised to aim for a representative day for calibrating the algorithm or to use more adaptive trigger settings, for example, via implementing dynamic algorithms [67], adjusting the intercept, which could account for shifts in autonomic regulation toward higher or lower levels of RMSSD. Furthermore, easier-to-handle algorithms might allow us to scale up the application of JITAIs by means of commercially available wearables.

Finally, although this research indicated that JITAIs triggered by nonmetabolic decreases of HRV are promising to intervene at the right moments and have convincing proximal effects, their long-term (distal) impact on physical and psychological well-being is yet unclear and calls for further research regarding their superiority relative to traditional intervention regimens. For example, it would be important to know if such brief interventions, when conducted regularly over a longer period, would have the potential to decrease risk for cardiovascular or mental diseases beyond the effects achieved by traditional intervention regimens.

### Conclusions

In this research, we demonstrated that applying brief, slow-paced breathing or mindful breathing interventions in everyday life via smartphones, triggered at moments of autonomic vulnerability, seems to stabilize mental health and cardiac autonomic function up to 10 minutes following the interventions, thus aligning with previous research conducted in traditional, standardized settings [10,19,20,23,36,59]. To the best of our knowledge, these are the very first microrandomized trials using an AddHRVr algorithm to automatically identify moments of psychophysiological vulnerability and trigger brief interventions (for a similar approach using physiological data, refer to the study by Hovsepian et al [68]). The findings are particularly important as they suggest that intervening at moments of compromised HRV could immediately boost cardiac autonomic function, and any kind of intervention could prove successful in restoring HRV up to 10 minutes to a certain degree. The findings were not restricted to a specific HRV metric but showed evidence toward a generalized increase in cardiac autonomic flexibility, thus suggesting clinical relevance. As speculated earlier, distraction or attentional deployment might be the core driver of these effects. In a similar vein, the general relieving effect on perceived stress and rumination independent of the trigger and type of intervention may signal a time-out effect, potentially restoring well-being.

The concept of JITAIs has raised considerable interest in recent years, and given the formidable growth and availability of wearables, digital mobile technologies, and algorithm developments, the field will continue to prosper [69]. However, research is yet in its infancy, and future studies need to convincingly demonstrate that JITAIs are superior to traditional intervention regimens and worth the tremendous effort in setting up algorithms sensitive and specific to certain psychophysiological states (eg, stress). In this respect, the adaptive part of JITAI needs is yet to be established. The proposed approach in this research did not adapt interventions to the specific needs but mainly followed the just-in-time concept.

### Statement of Relevance

Frequent episodes of adverse psychological states, such as stress, could impact health and well-being in the long run. Identifying such moments of vulnerability could be useful to directly counter such states via microinterventions to ameliorate their health impact. However, the usefulness of such interventions needs solid empirical research. In the 2 studies, we demonstrated that brief psychological exercises of 1-minute length, applied in moments of compromised autonomic flexibility (low nonmetabolic HRV), restored autonomic function and benefited psychological well-being. The findings suggest that brief psychological interventions can be implemented in daily life via wearables, possibly promoting mental and physical health longitudinally.

## Appendix

Multimedia Appendix 1 Schematic representation of the individually adjusted additional heart rate variability reductions algorithm based on the root mean square of successive difference and bodily movement.

Multimedia Appendix 2 Additional heart rate variability reduction algorithm calibration parameters derived from 12-hour baseline recordings in studies 1 and 2.

Multimedia Appendix 3 Intervention instructions for studies 1 and 2.

Multimedia Appendix 4 Multilevel regression predicting logarithmized root mean square of successive difference before, during, and after the slow-paced breathing intervention to 3 kinds of triggers (virtual additional heart rate variability reductions [AddHRVr], random, and AddHRVr). Estimates refer to raw b values (slopes).

Multimedia Appendix 5 Multilevel regression predicting logarithmized root mean square of successive difference before, during, and after intervention to 3 types of triggers (virtual additional heart rate variability reductions [AddHRVr], random, and AddHRVr). Estimates refer to raw b values (slopes).

## Abbreviations

AddHRVr: additional heart rate variability reductions
HF-HRV: high-frequency heart rate variability
HRV: heart rate variability
JITAI: just-in-time adaptive intervention
LF-HRV: low-frequency heart rate variability
ln: logarithmized
MET: metabolic equivalent
RMSSD: root mean square of successive differences
SDNN: SD of normal-to-normal
vmHRV: vagally mediated heart rate variability

## Data Availability

The datasets generated or analyzed during this study are available from the corresponding author on reasonable request.
