# Supplementary material for: Effectiveness of 2 Just-in-Time Adaptive Interventions for Reducing Stress and Stabilizing Cardiac Autonomic Function: Microrandomized Trials
Source: J Med Internet Res. 2025 Aug 7;27:e69582. doi: 10.2196/69582 (PMC12371293; doi:10.2196/69582)

*Fig. A1. Schematic representation of the individually adjusted AddHRVr algorithm based on RMSSD and bodily movement.* *Adapted with permission from Zeitschrift für Psychologie (2023), 231(4), 291–301. © 2023 Hogrefe Publishing www.hogrefe.com https://doi.org/10.1027/2151-2604/a000537*


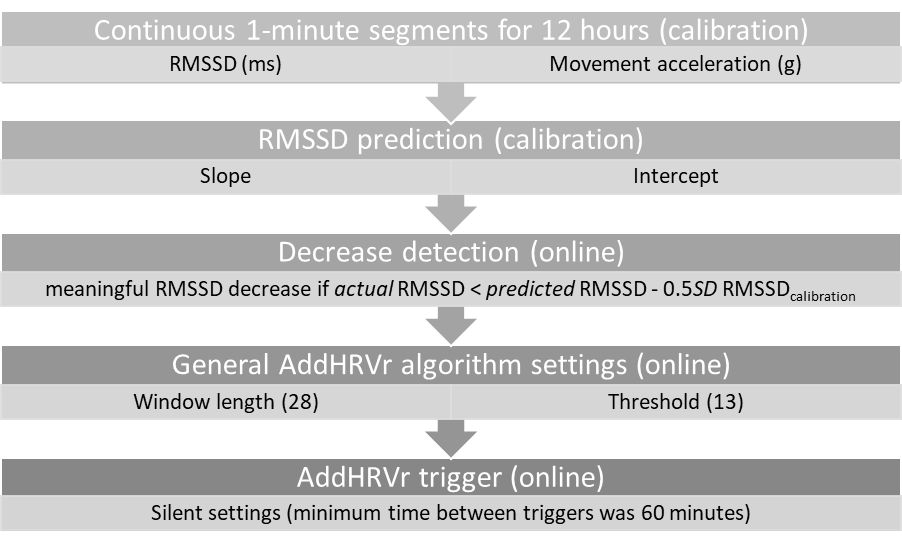

Supplement: Multimedia Appendix 1 [file jmir_v27i1e69582_app1.docx]
