# Supplementary material for: Effectiveness of 2 Just-in-Time Adaptive Interventions for Reducing Stress and Stabilizing Cardiac Autonomic Function: Microrandomized Trials
Source: J Med Internet Res. 2025 Aug 7;27:e69582. doi: 10.2196/69582 (PMC12371293; doi:10.2196/69582)
Supplement: Multimedia Appendix 2 [file jmir_v27i1e69582_app2.docx]

*Table A1. AddHRVr algorithm calibration parameters derived from 12h baseline recording in Study 1 and Study 2.*

|  | *M* | *SD* | *Max* | *Min* |
| --- | --- | --- | --- | --- |
| Intercept  Study 1  Study 2 | 47.55  46.94 | 18.98  22.29 | 101.66  121.87 | 20.87  10.72 |
| Slope  Study 1  Study 2 | -144.92  -125.23 | 100.67  79.75 | -14.26  -15.64 | -478.38  -344.55 |
| ½ *SD* (RMSSD)  Study 1  Study 1 | 9.70  9.54 | 4.66  5.06 | 23.79  28.40 | 3.97  2.94 |
| RMSSD  Study 1  Study 2 | 40.62  39.73 | 16.12  17.60 | 92.81  94.74 | 19.08  10.03 |
| Acceleration (g)  Study 1  Study 2 | 0.052  0.054 | 0.018  0.018 | 0.097  0.108 | 0.025  0.017 |
