# Supplementary material for: Effectiveness of 2 Just-in-Time Adaptive Interventions for Reducing Stress and Stabilizing Cardiac Autonomic Function: Microrandomized Trials
Source: J Med Internet Res. 2025 Aug 7;27:e69582. doi: 10.2196/69582 (PMC12371293; doi:10.2196/69582)
Supplement: Multimedia Appendix 3 [file jmir_v27i1e69582_app3.docx]

## Intervention instructions Study 1:

“Now follows a short breathing exercise during which you should inhale through your nose for 4 seconds and exhale through gently pressed lips for 6 seconds.” The exercise involving muscle contractions was announced as follows: “Now follows a short breathing exercise during which you should inhale through your nose for 4 seconds and exhale through gently pressed lips for 6 seconds. Please tense your dominant arm and make a fist while inhaling; release the tension while exhaling.”

## Intervention instructions Study 2:

Mindful breathing: “Now follows a breathing exercise. Try to adopt a relaxed yet upright position. The exercise lasts exactly one minute. For the next minute, focus only on your breath without judging or changing it. If your thoughts start to wander, simply acknowledge them without judgment and gently bring your attention back to your breathing. Start the intervention by clicking the checkmark in the top right corner. The exercise ends when the screen moves on to the next question.”

External cues: “Now follows an exercise. Try to adopt a relaxed yet upright position. The exercise lasts exactly one minute. For the next minute, focus only on a neutral stimulus in your surroundings without judging it. This could be, for example, a white wall or a chair. It is important that the stimulus is as neutral as possible, so I would advise against choosing a person. If your thoughts start to wander from the stimulus, simply acknowledge them without judgment and gently bring your attention back to your chosen stimulus. Start the intervention by clicking the checkmark in the top right corner. The exercise ends when the screen moves on to the next question.”
