# Supplementary material for: Effectiveness of 2 Just-in-Time Adaptive Interventions for Reducing Stress and Stabilizing Cardiac Autonomic Function: Microrandomized Trials
Source: J Med Internet Res. 2025 Aug 7;27:e69582. doi: 10.2196/69582 (PMC12371293; doi:10.2196/69582)
Supplement: Multimedia Appendix 5 [file jmir_v27i1e69582_app5.docx]

*Table A3. Multilevel regression predicting logarithmized RMSSD before (pre), during (intervention) and after intervention (post intervention) to three types of triggers (virtual AddHRVr, random, AddHRVr). Note that Estimates refer to raw b-values (slopes) and CI to confidence intervals.*

|  | **ln RMSSD** | | |
| --- | --- | --- | --- |
| *Predictors* | *Estimates* | *CI* | *p* |
| (Intercept) | 3.12 | 3.00 – 3.24 | **<.001** |
| Metabolic equivalents (METs) | -0.23 | -0.23 – -0.22 | **<.001** |
| Trigger (virtual vs. random) | 0.39 | 0.37 – 0.41 | **<.001** |
| Trigger (virtual vs. AddHRVr) | 0.02 | 0.01 – 0.04 | **.007** |
| Time (pre vs. intervention) | -0.02 | -0.04 – 0.00 | **.053** |
| Time (pre vs. post intervention) | 0.02 | 0.00 – 0.03 | **.025** |
| Trigger (virtual vs. random)  × time (pre vs. intervention) | 0.08 | 0.05 – 0.11 | **<.001** |
| Trigger (virtual vs. random)  × time (pre vs. post intervention) | -0.01 | -0.04 – 0.01 | .220 |
| Trigger (virtual vs. AddHRVr)  × time (pre vs. intervention) | 0.13 | 0.10 – 0.16 | **<.001** |
| Trigger (virtual vs. AddHRVr)  × time (pre vs. post intervention) | 0.07 | 0.05 – 0.10 | **<.001** |
| **Random Effects** | | | |
| σ^2^ | 0.21 | | |
| τ_00_ _participant_ | 0.18 | | |
| ICC | 0.47 | | |
| N _participant_ | 50 | | |
| Observations | 41609 | | |
| Marginal R^2^ / Conditional R^2^ | 0.141 / 0.543 | | |
